# New structure transistors for advanced technology node CMOS ICs

**DOI:** 10.1093/nsr/nwae008

**Published:** 2024-01-05

**Authors:** Qingzhu Zhang, Yongkui Zhang, Yanna Luo, Huaxiang Yin

**Affiliations:** Integrated Circuit Advanced Process R&D Center, Institute of Microelectronics of Chinese Academy of Sciences (IMECAS), Beijing 100029, China; State key Lab of Fabrication Technologies for Integrated Circuits, Institute of Microelectronics of Chinese Academy of Sciences, Beijing 100029, China; Integrated Circuit Advanced Process R&D Center, Institute of Microelectronics of Chinese Academy of Sciences (IMECAS), Beijing 100029, China; State key Lab of Fabrication Technologies for Integrated Circuits, Institute of Microelectronics of Chinese Academy of Sciences, Beijing 100029, China; Integrated Circuit Advanced Process R&D Center, Institute of Microelectronics of Chinese Academy of Sciences (IMECAS), Beijing 100029, China; School of Integrated Circuits, University of Chinese Academy of Sciences, Beijing 100049, China; Integrated Circuit Advanced Process R&D Center, Institute of Microelectronics of Chinese Academy of Sciences (IMECAS), Beijing 100029, China; State key Lab of Fabrication Technologies for Integrated Circuits, Institute of Microelectronics of Chinese Academy of Sciences, Beijing 100029, China; School of Integrated Circuits, University of Chinese Academy of Sciences, Beijing 100049, China

**Keywords:** transistor, CMOS, GAAFET, CFET, 3DS FET

## Abstract

Over recent decades, advancements in complementary metal-oxide-semiconductor integrated circuits (ICs) have mainly relied on structural innovations in transistors. From planar transistors to the fin field-effect transistor (FinFET) and gate-all-around FET (GAAFET), more gate electrodes have been added to three-dimensional (3D) channels with enhanced control and carrier conductance to provide higher electrostatic integrity and higher operating currents within the same device footprint. Beyond the 1-nm node, Moore’s law scaling is no longer expected to be applicable to geometrical shrinkage. Vertical transistor stacking, e.g. in complementary FETs (CFET), 3D stack (3DS) FETs and vertical-channel transistors (VFET), for enhanced density and variable circuit or system design represents a revolutionary scaling approach for sustained IC development. Herein, innovative works on specific structures, key process breakthroughs, shrinking cell sizes and design methodologies for transistor structure research and development are reviewed. Perspectives on future innovations in advanced transistors with new channel materials and operating theories are also discussed.

## Teaser

The transistor structure innovations are critical for advanced IC development. Beyond the evolution of up-to-date GAAFET to traditional FinFET, more advanced transistors like complementary FETs (CFETs), 3D stack (3DS)-FETs and vertical-channel transistors are developing for enhanced integration density and circuit design capability to sustain the scaling of Moore’s law.

## INTRODUCTION

The invention of the transistor in 1947 and the ongoing development of integrated circuits (ICs) since 1958 have inspired dramatic improvements in information technology (IT) and computing [1], including the evolution from mainframe computers to personal computers, and the development of the Internet, mobile communications and artificial intelligence, which have had a global impact on people’s lives, culture and society [2]. ICs follow the scaling principle known as Moore’s law [3] and serve as the critical engine that drives IT functionality and computing efficiency. Their versatility stems directly from the decreasing size and increasing density of transistors in ICs that are enabled by technological breakthroughs in carrier transport control, largely in silicon (Si)-based semiconductor devices [4,5]. These devices are mainly based on complementary metal-oxide-semiconductor (CMOS) technology, which is used to manipulate the electrical conductance of both *n*-type (electron) and *p*-type (hole) carriers simultaneously. CMOS transistors offer several advantages over earlier bipolar junction transistors and single carrier-type metal-oxide-semiconductor field-effect transistors (MOSFETs), demonstrating higher static power efficiency and greater integration density.

**Figure 1.**
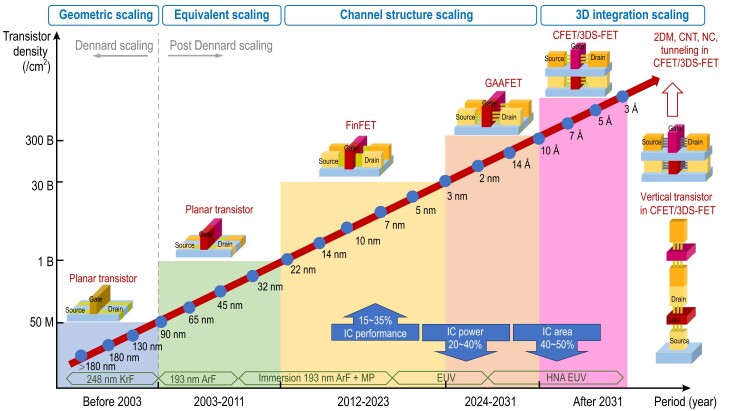
Development history of and new trends in MOSFETs with differing transistor structures and scaling stages.

Moore’s law predicted that the number of integrated CMOS transistors per square centimeter would double every two years, with this number sometimes doubling every 18 months. Originally, as shown in Fig. 1, device development was consistent with Robert Dennard’s scaling guidelines [6] for shrinking feature sizes (i.e. process node scaling) while also increasing device currents and reducing the operating voltages. Subsequently, this led to critical specification improvements in terms of performance, power and area (PPA), which meant that ICs evolved with an almost fixed gain rate of 30%–50% in later years, along with falls in fabrication cost per transistor in ICs [7].

Dennard’s scaling is also known as conventional geometric scaling and this type of scaling smoothly stimulated exponential growth in the IC industry until 2003. For the ICs that were developed beyond the 90-nm node, power scaling has replaced traditional geometric scaling and has become the predominant scaling factor for CMOS ICs. A series of innovative technologies based on new process materials, including embedded silicon-germanium (SiGe) source/drain and high-κ/metal gates (HKMGs), have been implemented to enhance carrier conductance via channel strain engineering, while also improving gate control and reducing leakage currents in MOSFETs through continuous scaling of the equivalent gate dielectric thickness (EOT) with shorter gate lengths (*L_G_*) [8]. Rather than geometric scaling, the power consumption scaling limit has become the dominant factor in IC scaling, and thus CMOS technology has entered the post-Dennard-scaling era.

As ICs continued to be developed beyond the 22-nm node, as shown in Fig. 1, further scaling of the transistor *L_G_* using singular and planar gate structures was no longer sufficient to maintain the electric field strength and switch off the transistor effectively. The nonplanar, self-aligned double-gate transistor structure called the fin field-effect transistor (FinFET) was introduced to improve control of the channel conductance and continue *L_G_* scaling, while also providing enhanced operating currents within the same device area (‘footprint’) by increasing the number of effective gate controls via a multi-gate structure on the three-dimensional (3D) Si fin channel structure [9]. The FinFET and its successor, the tri-gate transistor, which has triple-gate controls that partially surround the 3D fin channel on bulk Si, were combined with the integration of SiGe strain engineering and HKMG technologies to provide a core device solution to maintain advanced CMOS IC scaling down to the 3-nm node; this node belongs to the channel structure scaling era [10]. At this stage, the fabrication cost per transistor was rising dramatically, but was covered completely by the increased market for advanced chips. Therefore, the economic effect, as one of the most important driving forces, still allowed transistors to be scaled down.

As the transistor *L_G_* continued to scale and the contacted gate pitch (CGP), or contacted poly pitch, representing the smallest space between the gate electrodes of neighboring transistors, continued to shrink, transistor structures beyond FinFETs were explored extensively to overcome more serious challenges related to power consumption, performance degradation and process variability in ultra-large-scale IC manufacturing. After attempts to innovate and transform FinFETs (e.g. the taller-fin FinFET and the high-mobility channel FinFET), the most revolutionary improvement came in the form of gate-all-around (GAA) architectures, where the channels are often made from nanoscale Si films with vertically stacked multi-layers, which are commonly called stacked nanowires (NWs) or nanosheets (NSs), and are all surrounded by gate dielectrics and gate electrodes [11–13]. GAA devices such as NS gate-all-around FETs (GAAFETs) with stacked NS layers showed obviously improved electrostatic performance when compared with FinFETs and enabled further *L_G_* scaling and increased drive currents within the same footprint, which also enhanced the design flexibility for IC layouts. Furthermore, the NS GAAFET was developed for state-of-the-art IC manufacturing and is compatible with existing fabrication processes for FinFETs. The NS channel direction is lateral and the channel is only formed selectively under the transistor gate regions, while the semiconductor portions within the source/drain (SD) regions are almost identical to the corresponding regions formed in FinFETs.

Beyond GAAFETs, as shown in Fig. 1, scaling of the transistor *L_G_* approaches the insurmountable limitations caused by short-channel effects (SCEs) (∼10-nm channel length limit) because no more gate controls can be added to strengthen control of the channel conductance. However, scaling of the standard circuit (SDC) and static random-access memory (SRAM) cells to increase the transistor integration density became the focus of advanced IC development. One potential structure involves insertion of an isolated dielectric layer within stacked NSs, called a forksheet FET, to tighten the *n*-type and *p*-type transistor spacing, which may then shrink the cell layout from six tracks (6T) to 5T or 4.5T. Another novel technology that involves an isolated dielectric is the buried power rail (BPR). However, another more groundbreaking structure, called the complementary FET (CFET) or the 3D stack FET (3DS FET), was achieved by stacking a transistor of one channel type on top of another transistor for almost half the CGP; subsequently, a four-track layout or less could then be obtained. Introduction of CFETs does not deliver stronger channel electrostatic control, but rather provides enhanced performance and area benefits for various integration approaches and use of different channel materials, transistor structures and circuit design layouts for the top- and bottom-tier devices [14,15].

In addition, the vertical-channel GAA transistor (or vertical transistor) with its 3D stackable structure is expected to enable further integration density enhancements in the future. Furthermore, a series of breakthroughs have been explored in transistor operation theory, including the tunneling transistor, the Dirac source and negative capacitance, to surpass the fundamental Boltzmann tyranny, and also in transistor channel materials, including pure germanium (Ge), carbon nanotubes (CNTs), two-dimensional materials (2DMs) and amorphous metal-oxide semiconductors (AOSs, e.g. indium gallium zinc oxide (IGZO)) for higher carrier mobility, thinner film thickness or better 3D stackable ability. These groundbreaking material properties and carrier conductance theories have been utilized to enhance transistor performance and power efficiency when compared with traditional Si devices. It is predicted that structural design innovations at the transistor level are most likely to turn to innovations at the circuit or chip level, and the implementation of new semiconductor materials will have a greater impact on transistor performance acceleration to aid in development of more advanced ICs with higher computing power and efficiency [16]. Additionally, innovations in integrated storage-calculation technology [17], chip-level monolithic 3D integration and even quantum computing [18] will spur on the emergence of more revolutionary IC and computing systems.

## BENEFITS AND CHALLENGES FOR TRANSISTOR STRUCTURE INNOVATIONS

The main goal in transistor structure innovation is to maintain electrostatic integrity in the Si channel to sustain either *L_G_* or CGP scaling and thus increase the number of transistors that can be integrated into a single chip for enhanced the PPA [19]. This concept is expressed theoretically in the equations


(1) \begin{eqnarray*}
{\lambda _{\rm MOSFET} = \sqrt{\frac{\epsilon _{\rm Si}}{n\epsilon _{\rm ox}}t_{\rm Si}t_{\rm ox}}}, \qquad n=1,\dots ,4,
\end{eqnarray*}

(2) \begin{eqnarray*}
\lambda _{\rm GAAFET} = \sqrt{\frac{2\epsilon _{\rm Si}t_{\rm Si}^2\ln (1+{{2t_{\rm ox}^2}/{t_{\rm Si}})} +\epsilon _{\rm ox}t_{Si}^2}{16\epsilon _{\rm ox}}t_{\rm Si}t_{\rm ox}},
\end{eqnarray*}

where lambda (λ) is the natural scaling length of the MOSFET, and its value should be less than 1/4 of the *L_G_* or *L*_eff_ (electrical effective *L_G_*). Among the other parameters, *n* represents the number of gate controls, ϵ_Si_ and ϵ_ox_ are the dielectric constants, and *t*_Si_ and *t*_ox_ are the thicknesses of the Si channel and the gate insulator, respectively. The electrostatic integrity of the transistor is quantified using two key metrics: the subthreshold slope (*SS*) and the drain-induced barrier lowering (*DIBL*). Both the *SS* and the *DIBL* are degraded as the *L_G_* is scaled, as expressed in


(3) \begin{eqnarray*}
{\rm SS} = 60\bigg (1+\frac{n\lambda _{\rm MOSFET}^2}{t_{\rm Si}}\bigg ),
\end{eqnarray*}

(4) \begin{eqnarray*}
{\rm DIBL} = \alpha {\frac{\lambda _{\rm MOSFET}^2}{{L}_{\rm eff}^2}}\bigg (1+\frac{x_{j}^2}{{L}_{\rm eff}^2}\bigg ){{V}_{\rm DS}},
\end{eqnarray*}

where α and *x_j_* are the coefficient and the junction depth of the SD region in the MOSFETs. Increasing the gate control number (*n*) significantly reduces the value of λ, and, as a result, degradation of both the *SS* and the *DIBL* is suppressed meaningfully and the transistor’s scaling ability is extended. Simultaneously, the operating voltage of the transistor with multiple gate electrodes is reduced and the transistor off-state current is also reduced for lower *SS* and *DIBL* values, which are beneficial for power reduction. The important structural and process parameters for various transistor structures are summarized in Table 1.

**Table 1.** Key structure and process parameters for various structure transistors.

Transistor structures		Planar			CFET or	Vertical
and technology		transistor	FinFET	GAAFET	3DS FET	transistor
1	Process node	∼22 nm\20 nm	22–3 nm	3–1 nm	∼1 nm	Beyond
2	CGP	>80 nm	80–48 nm	48–38 nm	40–36 nm	80–48 nm
3	Gate length	>24 nm	24–14 nm	14–10 nm	12–10 nm	>20 nm
4	EOT	>0.9 nm	∼1.0 nm	∼1.0 nm	<1.0 nm	∼1.0 nm
5	SDC tracks	>7.5 T	7.5–6 T	6–5 T	<5 T	<5 T
6	Transistor density	<0.5 B cm^−2^	0.5–30 B cm^−2^	30–300 B cm^−2^	>300 B cm^−2^	>300 B cm^−2^
7	Power voltage	<0.9 V	0.9–0.7 V	0.7–0.6 V	0.7–0.6 V	0.9–0.6 V
8	Performance factor per footprint	1	2–2.5	3–6	6–12	5–10
9	Scaling factor	>10 nm	∼4 nm	∼3 nm	∼3 nm	∼3 nm
10	Gate control per channel	1	2–3	4 or all round	4 or all round	4 or all round
11	Fine lithography per footprint	i-193 nm ArF	MP, EUV	MP, HNA EUV	MP, HNA EUV	i-193 nm ArF, MP
12	Key process	Strain, HKMG	Strain, HKMG,	Strain, HKMG,	HKMG,	HKMG,
	technology		DTCO	DTCO, BSPDN	STCO, BSPDN	DTCO
13	Channel doping	SSRW, peak	Uniform	Uniform	Uniform	Uniform
		5 × 10^18^ cm^−3^	3 × 10^16^ cm^−3^	1 × 10^16^ cm^−3^	1 × 10^16^ cm^−3^	1 × 10^16^ cm^−3^
14	Channel material	Si	Si, SiGe	Si, SiGe	Si, SiGe, Ge,	Si, SiGe,
					CNT, 2DM, AOS	III-V
15	Channel carriers	DD	Q-Ballistic	Ballistic	Ballistic	Ballistic
	conductance				tunneling, NC	tunneling, NC

Furthermore, as the channel profile is transformed from planar Si to a 3D Si fin profile, the effective width for carrier transport in the channel (performance factor per unit footprint) between the transistor SD electrodes is doubled or even tripled in FinFETs and is multiplied even more in GAAFETs and CFETs; this amplifies the saturated on-state current of the transistor within the same footprint. For stronger gate control in FinFETs and GAAFETs, channel doping in the Si channel is reduced greatly and less carrier scattering occurs, which changes the transistor conductance from the surface-inversion mode to the volume-inversion mode, where parameters such as the threshold voltage (*V*_T_) can be suppressed and the minimum operating voltage in large-scale SRAM arrays is reduced; this is beneficial for PPA gain [20].

The 3D channel transistor and the 3D stacking transistor also enable novel process innovations, including the BPR and the backside power delivery network (BS PDN), which may further reduce the SDC and SRAM cell areas and provide higher transistor densities [21]. CFETs consist of two-tier transistors integrated into a single device and offer an opportunity for the introduction of innovative semiconductor materials beyond traditional Si into mainstream IC manufacture, e.g. Ge on Si, 2DMs on Si and CNTs on Si through selection of the sequential 3D integration process; this process may obtain the best carrier conductance between *n*-type and *p*-type transistors in CMOS ICs and also expand PPA gain in advanced ICs by developing new transistor structures and processes.

However, to transition successfully from planar transistors to FinFETs, from FinFETs to GAAFETs and from GAAFETs to CFETs or vertical transistors in advanced ICs, a series of innovations will be required, including key device fabrication and processing breakthroughs, cell area reduction and advances in the design methodology. The transistor structures should be compatible with previous nodes and the critical processing methods and parameters must be developed carefully. The HKMG and strain technologies should also be upgraded for advanced transistor structures. A design technology co-optimization (DTCO) approach for advanced FinFETs and GAAFETs must be established to optimize the PPA for complex structures and processes. For favorable integration of more complex devices such as CFETs with BPR, BS PDN or monolithic 3D ICs and heterogeneous integration systems, a system technology co-optimization (STCO) [22] method must be established for sustained development of advanced ICs with enhanced functionality, energy efficiency and power.

## TRANSITION FROM FINFET TO GAAFET

### Advanced FinFET and innovations

The FinFET is currently the fundamental transistor structure in mainstream IC manufacturing and has been widely used in several technology generations, from the 22-nm node to the 3-nm node. As FinFET scaling continues for higher device density and improved PPA, 3D Si fins are becoming thinner and taller, which is causing reduced carrier mobility, large-scale random doping fluctuations and major *V*_T_ differences between the individual top and bottom devices [23]. A high-mobility channel using SiGe fins has been introduced into 5-nm FinFETs [24] to mitigate performance degradation in scaled *p*-type FinFETs. Contact on gate, double diffusion breaks (DDBs) and single diffusion breaks (SDBs) are structural design concepts used for continuous scaling of SDC and SRAM cells. New materials such as Ru or Co metals and new structures like BS PDNs have also been introduced to enhance the back-end interconnection performance between transistors.

Some structural innovations are being developed to extend the lifetime of the FinFET manufacturing process. To improve the device’s gate control ability and reduce channel leakage in the sub-fin region, the FinFET on silicon-on-insulator (SOI) structure has been introduced by IBM and CEA-Leti [25]. Implementation of more gates using the Π-gate and Ω-gate structures in FinFETs, along with the special scalloped FinFET, is also being investigated [26]. To suppress the parasitic channel effect (PCE) that occurs beneath the fin on a bulk Si wafer completely, a novel FinFET with a fully isolated fin channel, which was achieved using bottom notch-etch and partial oxidation processes, has been presented by IBM and the Institute of Microelectronics of the Chinese Academy of Sciences (IMECAS) [27,28].

### GAAFET integration process method

**Figure 2.**
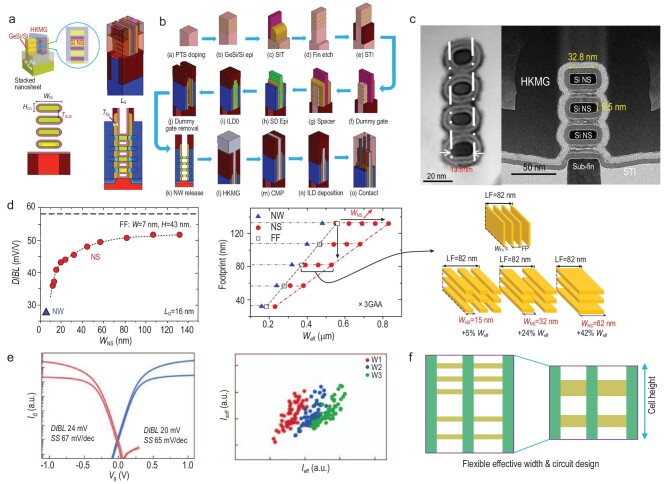
(a) Typical structure and (b) fabrication flow for the stacked NS GAAFET. (c) Cross-sectional transmission electron microscopy (TEM) images of a stacked Si NW GAAFET and a stacked Si NS GAAFET. (d) Comparison of gate controllability, performance and effective gate width (*W*_eff_) among FinFETs, Si NW GAAFETs and Si NS GAAFETS. (e) Performance and circuit improvements in stacked Si NS GAAFETs. (f) Variable effective width and circuit design for stacked Si NS GAAFETs.

Around the 3-nm node, even when using advanced processing technologies, the extremely scaled FinFET suffers from SCEs and performance degradation because of its ultra-thin and high channel dimensions. GAAFETs are expected to be selected for use as next-generation transistors in advanced ICs. The typical GAAFET structure and its fabrication flow are shown in panels (a) and (b) of Fig. 2, respectively.

Early fabrication approaches for the GAA device used an NW-first scheme based on selective etching and oxidation between two large SD landing pads, but this approach is not compatible with existing FinFET processes [29]. An NW-last scheme in a replacement metal gate (RMG) module based on an HKMG FinFET was proposed by Ma at IMECAS in 2015 [30], where the Si NW was released by dilute HF etching with the support of the spacers and zero-level interlayer dielectric. Stacked Si NW-last GAAFETs with GeSi/Si superlattice stacks offer a more feasible solution and the gate control capabilities of the transistor with stacked Si NW diameters of 8 nm in the RMG module realized by a selective release process were first demonstrated by IMEC in 2016, based on their bulk Si HKMG-FinFET process [31]. They demonstrated a minimum *SS* of 65 mV/dec and a *DIBL* of 42 mV/V for their CMOS devices with a physical *L_G_* of 24 nm, which was better than corresponding FinFET devices. Vertically stacked NW GAAFETs with high-mobility SiGe channels were also presented by IMECAS in subsequent years, as shown in Fig. 2(c) [32].

In 2017, IBM reported stacked three-layer Si NS GAAFETs with increased drive currents and greater process compatibility with mainstream manufacturing [33]. These devices contained vertically stacked flat and thin Si NSs located in the transistor channel and demonstrated strong SCE immunity, like that of the NW GAAFET, and also offered a greater effective transistor channel width because of the number of stacked Si NSs and the increased width (Fig. 2(d)). Approximately 80% of the processes used were the same as those used for the conventional HKMG-last FinFET, except for the parasitic channel suppression implantation, GeSi/Si superlattice epitaxy, inner spacer preparation and channel release steps. Before forming the epitaxial multi-layer SiGe/Si superlattice stacks, doping must be performed to suppress the parasitic sub-fin channels. The 3D fin, shallow trench isolation (STI), dummy gate and spacer formation steps are similar to those used for the FinFET. The inner spacers are formed by SD fin etching, SiGe cavity etching and spacer etching with advanced reactive ion etching (RIE) and a new etching method as well as a conformal filling with thin SiN_*x*_ deposition. The SiGe epitaxy process with *in situ* doping is then conducted to reduce the parasitic resistance of the SD region and apply strain in the channel. After removal of the α-Si dummy gate, the Si NW/NS channels are formed by selective removal of SiGe. The subsequent HKMG, contact and back-end-of-line (BEOL) processes for Si GAAFETs are the same as those used to prepare FinFETs.

More recently, Samsung reported stacked three-layer Si NS GAAFETs with 12-nm *L_G_*s and 48-nm CGP for final industrialized manufacture [34]. IMECAS also presented a vertically stacked Si NS GAAFET fabricated by deep ultraviolet lithography, as shown in Fig. 2(c). The increased Si NS width increases the operating current while also maintaining nearly perfect gate control. The Si NS GAAFETs also have a broad allowable effective width that can enable more flexible and compact circuit design (Fig. 2(f)). Samsung fabricated the world’s first GAA 3-nm foundry platform technology (SF3) with the novel multi-bridge-channel MOSFET (MBCFET™) process and achieved a 22% speed gain, a 34% power gain and a 79% logic area reduction when compared with their previous 4-nm FinFET platform (Fig. 2(e)).

### Key GAAFET process technologies

**Figure 3.**
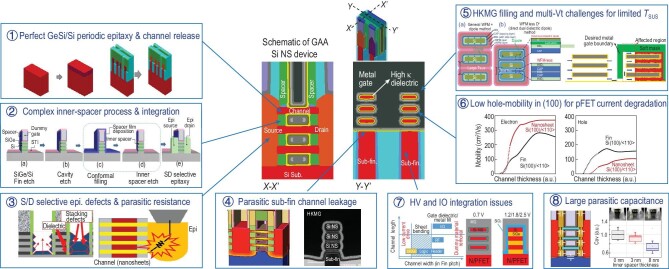
Critical process challenges for stacked Si NS-GAA device fabrication and the corresponding process technologies.

Although Si NS GAAFETs provide significant advantages in terms of gate control, overall performance and circuit design flexibility, the fabrication process still involves numerous challenges, as illustrated in Fig. 3. They include processes for high-quality GeSi/Si superlattice periodic epitaxy and channel release, and a complex inner spacer module, SD-selective epitaxial defects, parasitic sub-fin channel leakage and HKMG filling, along with multiple *V*_T_ tuning challenges because of limited NS spacing (*T*_SUS_), low hole mobility in the (100) orientation, high voltage (HV) and input/output (IO) integration, and high parasitic capacitance during AC operation.

The SiGe/Si superlattice structure is usually grown epitaxially on Si substrates by reduced-pressure chemical vapor deposition. To obtain high-quality GeSi/Si superlattice thin films with accurate thicknesses and Ge components, the epitaxial process is generally performed at temperatures of less than 650 ^○^C, and the film defects are controlled precisely. When removing the SiGe sacrificial layer in the RMG, it is necessary to have a sufficiently high selectivity ratio (over 100 : 1 for SiGe : Si) to avoid damage in the Si channel. The selective etching of SiGe layers is mainly achieved by dry etching, HCl gas etching or wet etching.

The inner spacer was designed to reduce the parasitic capacitance between the gate and SD electrodes in stacked NS GAAFETs, but the preparation of the inner spacer process is relatively complex, containing more than 10 main steps. Furthermore, the most critical and challenging processes are vertical selective isotropic etching of the SiGe fin, anisotropic selective etching of the SiGe to the Si for cavity formation, the conformal ultra-thin dielectric filling process and anisotropic selective etching of the inner spacers with a selectivity ratio of more than 100 : 1 for both the Si : SiO_2_ and Si : Si_3_N_4_ structures. Li *et al.* [35] obtained a uniform inner spacer by using a high-precision controllable silicon nitride inner spacer structure that was prepared using an inductively coupled plasma tool and a new gas mixture of CH_2_F_2_/CH_4_/O_2_/Ar; they also developed quasi-atomic layer etching techniques with an accuracy of ∼0.3 nm/cycle. In addition, because of the poor-quality conductance pathway between the stacked NS channels and SD electrodes formed simultaneously by complex selective SiGe or Si epitaxy processing of the bottom substrate and the separated multi-layer Si NSs, high parasitic resistances often occur, causing the operating currents to degrade [36].

The ‘fat-fin’ effect is unique to Si NS GAAFETs, where process non-idealities can increase the high sub-fin leakage and capacitance below the Si NS channel. Zhang *et al.* [37] systematically investigated the influence of ground-plane (GP) doping on the leakage induced by the fat-fin effect through experiments and simulations. The subthreshold characteristics of the *n*-type devices were greatly improved by increasing the GP doping doses, whereas the corresponding *p*-type devices initially improved and then deteriorated with increasing GP doping doses, demonstrating their optimal electrical characteristics at GP doping concentrations of approximately 1× 10^18^ cm^−3^. Gu *et al.* [38] proposed a new etching technique that narrowed the sub-fin with little increase in the processing cost that suppressed the PCE. Their proposed sub-fin design demonstrated a 70% reduction in the sub-channel gate-induced drain leakage and a 20% increase in the on-off current ratio (*I*_ON_/*I*_OFF_), along with an improvement in the *SS*. In addition, a full bottom dielectric isolation approach was demonstrated for Si NS-GAA device structures by Zhang from IBM. First, a Ge_*x*_Si_1−*x*_ sacrificial layer with high Ge content was designed in an epitaxial procedure; this layer was then selectively removed in a subsequent process and filled with a dielectric material by atomic layer deposition (ALD) to form a global isolation layer on the bottom of the structure. They reported a reduction of approximately 2 orders of magnitude in the off-state leakage current, a 4.4% reduction in the parasitic capacitance and a 4.6% improvement in *F*_max_ [39].

The HKMG module is among the most challenging components of the Si NS-GAA structure because of its limited fin pitch and limited *T*_SUS_. Interface dipole engineering provides a volume-less technique for effective work function tuning that is unlike the techniques used in conventional work function metal (WFM) engineering. Generally, dipoles form in a layer that is deposited on the high-κ layer and require high-temperature drive-in annealing, which can cause both SD stress release and reliability issues. Yao *et al.* [40] reported 7(N) + 7(P) multi-*V*_T_s on GAA Si NS devices that were fabricated using ultra-thin low-temperature hybrid ALD *in situ* La-/Al-dipole approaches and both *n*- and *p*-type FETs have achieved seven *V_T_*s with high uniformity and differentiation.

Additionally, the hole mobility of stacked Si NS GAAFETs when using the 〈100〉 crystal orientation is far lower than their electron mobility. To improve their hole mobility, high-mobility SiGe- or GeSn-stacked channels have been introduced into *p*-type devices [41]. Mochizuki from IBM and coworkers [42] proposed a SiGe channel for *p*-type-stacked NS GAAFETs produced by channel trimming of 1–2 nm per side and conformal SiGe clamping layer epitaxial growth of 2–3 nm. Furthermore, a Si capping layer was applied as a passivation layer and *D*_it_ was reduced by one order of magnitude. Because of their limited *T*_SUS_, it is feasible to use traditional FinFETs with multi-layer SiGe/Si structures for high voltage and input/output devices, but hybrid integration with selective channel release and use of various dielectric thicknesses remains a key challenge.

### Innovations on GAA devices

**Figure 4.**
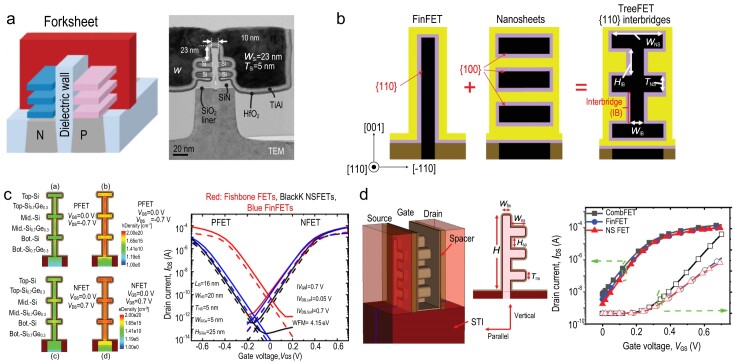
(a) Three-dimensional schematic and TEM image of a forksheet FET. (b) Cross-sectional structure of a TreeFET and simulated carrier densities in its channels. (c) Schematics of fishbone FETs fabricated using the gate-first process on bulk Si and simulated device transfer curves. (d) Schematics of a CombFET and simulated device transfer curves.

To increase the density of the integrated transistors, further developments in both manufacturing processes and device structures are required. Forksheet FETs, as shown in Fig. 4(a), were developed at IMEC to provide tighter spacing between the *n*- and *p*-type FETs in both SDC and SRAM cells by inserting one dielectric layer into the stacked NSs, which may enable circuit track shrinkage with little modification of the existing manufacturing processes [43]. However, the forksheet FET has two major drawbacks, comprising degraded gate control caused by the semi-around gate structure of the NS channel adjacent to the insulating sidewall dielectric on one side, and worsening distribution fluctuations in the device’s electrical characteristics caused by the dielectric insertion process. To enhance the device driving current while maintaining a limited footprint and limited channel height, both tree and fishbone FET structures (panels (b) and (c) of Fig. 4, respectively) have recently been proposed that combine vertically stacked NS channels with nano-fin-shaped interbridge channels. Specifically, Tu *et al.* [44] proposed an epitaxial doping scheme to reduce the *V*_T_ differences caused by using different materials, specifically between the NSs and the interbridge channels. Cao *et al.* [45,46] proposed and demonstrated one feasible fabrication approach for fishbone FETs using channel-first and single WFM processes. Li *et al.* [47] proposed a novel comb-like channel device (CombFET), as shown in Fig. 4(d), which combines FinFET and NS-FET geometries in its channel region and may offer higher operating currents.

## TRANSITION FROM GAAFET TO CFET

**Figure 5.**
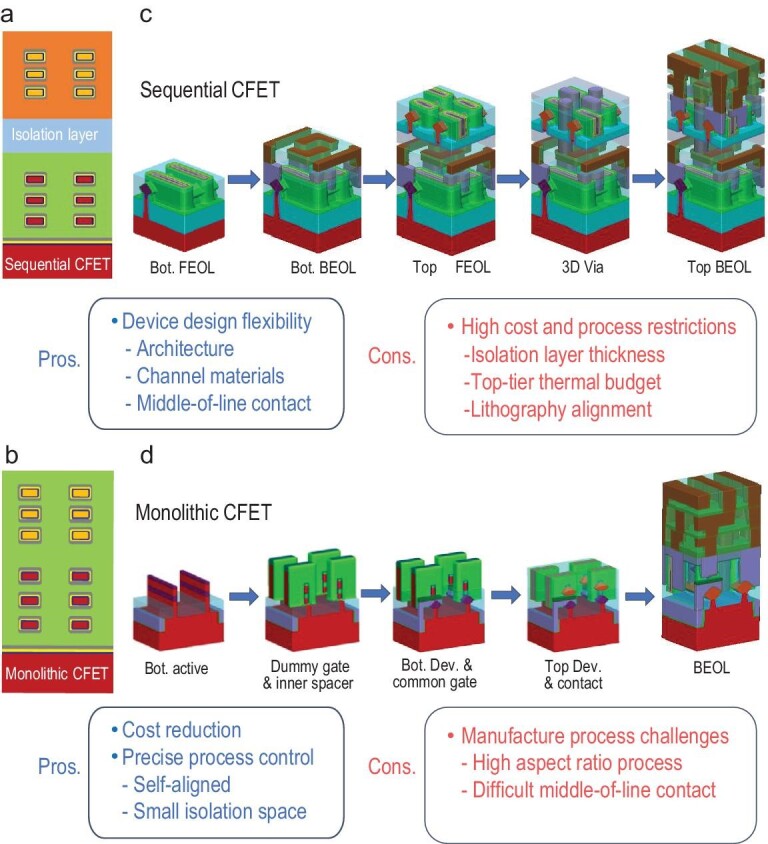
(a) Cross-sectional structure of the sequential CFET. (b) Cross-sectional structure of the monolithic CFET. (c) Schematic of the process flow and the key modules for sequential CFET fabrication. (d) Schematic of the process flow and the key modules for monolithic CFET fabrication. The advantages and disadvantages of the sequential CFET and the monolithic CFET are also shown.

Beyond GAAFETs, CFETs are showing promise for scaling toward the 1-nm node. As shown in Fig. 5, the CFET consists of vertically integrated *n*- and *p*-type FETs. In advanced process nodes, reduction of the SDC and SRAM cell heights is mainly limited by the *n*-to-*p* spacing requirements. CFETs avoid the limitations of *n*-to-*p* spacing by translating their horizontal *n*-to-*p* spacing into the vertical direction, thus reducing the cell height to four or even three tracks [48]. Reduction of the SDC cell height in CFETs is also dependent on application of the BS PDN. The main connectivity schemes include the through- silicon via in the middle of the line, the BPR and the backside contact structure [49]. These three BS-PDN options all involve a trade-off between PPA gain and process integration complexity. In CFET cells, the vertically stacked devices can provide more space to optimize the effective channel width of the device, thereby improving the device drive current. As a result of the extension of the CFET geometry in the vertical direction, the interconnections are changed significantly. In the past, IC manufacturing processes could be divided into front-end-of-the-line (FEOL), middle-of-the-line (MOL) and BEOL processes. The CFET is a full 3D structure in the circuit, and its interconnections are also expanded into a 3D circuit on the transistor or block level. During CFET manufacture, the FEOL, MOL and BEOL processes intersect and sometimes merge. Therefore, the huge benefits of unit area reduction provided by CFETs come at the cost of more complex processes, including vertical interconnection technology, active channel vertical stacking technology, SD vertical stacking technology and DTCO technology.

### CFET integration process method

Based on the manufacturing processes and the structural features of CFETs, there are two device types: sequential and monolithic CFETs. The main difference between these two structures is whether the manufacturing processes for the top and bottom devices are highly coupled. Panels (a) and (b) of Fig. 5 show the cross-sectional structures of sequential and monolithic CFETs, respectively. In the sequential CFET, there is a thick isolation medium between the bottom- and top-tier devices, and there may be one or multiple interconnected metal layers between them. Furthermore, the source, drain and gate electrodes are relatively far apart and require 3D interconnections to form a 3D circuit. In a monolithic CFET, the top- and bottom-tier devices are closer together and usually have a common gate structure, which means that the gates of the top and bottom devices are connected and formed during the same process step.

Figure 5(c) shows a schematic diagram of the sequential CFET fabrication process. First, traditional IC manufacturing processes are used to produce the bottom transistors and the interconnect metals. Isolation of the interconnect metals is then necessary before production of the top layer begins. Because the bottom layer has already undergone BEOL processing, the 3D isolation process requires metal ion contamination to be controlled to ensure that the top-layer devices are not affected by metal ion diffusion. The first step in production of the top-layer devices is vertical stacking of the active layers. The active layers can include materials with different crystal orientations to the bottom layer or even different types of materials, depending on the circuit type and the performance optimization requirements. At this point, to ensure the stability and the reliability of the underlying bottom-tier devices, the process flow for the top level must be controlled to within a specific thermal budget range [50]. The top and bottom devices are electrically interconnected using 3D vias. These 3D vias must pass through both the active layer and the isolation layer and also have a high aspect ratio. They may form high-precision interconnections with granularity between the top and bottom layers to suppress any obvious overlay bias between different chips, similar to advanced 3D package technology. Because the fabrication processes for the top and bottom layers are separate, the layers can be optimized separately to use optimal process conditions and design methods. Furthermore, separation of the processes for the upper and lower layers potentially allows for adoption of mature planar circuit processes.

Figure 5(d) shows a schematic diagram of the monolithic CFET fabrication process, which is relatively complex. Given the high degree of coupling between the manufacturing processes for the top and bottom devices, the manufacturing process cannot simply be divided into the top- and bottom-tier device processes. At the same time, the mature process methods that this new structure can use are limited, and several new process modules must be developed to fabricate this structure. As shown in Fig. 5(d), the most important process modules include the active layer vertical stacking, SD vertical stacking, channel release, separate gate formation and top/bottom SD stacking modules. Unlike the sequential integrated CFETs, vertical stacking of the active layers in a monolithic CFET represents the first step in the manufacturing process. The epitaxial process, with its advantages of low cost and reliable formation of high-quality single-crystal structures, is usually implemented. At this point, a sacrificial epitaxial layer is required between the top channel and the bottom channel. Subsequently, similar to the traditional HKMG transistor process, dummy gates and spacers must be manufactured first. Then, to improve contact efficiency between the top and bottom layers and utilize the interconnection resources fully, it is necessary to form the bottom SD electrodes first. The top/bottom SD vertical stacking process also relies on isolation technology for electrical insulation in the limited vertical spaces. For introduction of the metal materials, isolation methods and strict contamination control processes are equally important. Unlike conventional planar circuit processes, in which the SD electrodes are extracted directly, these processes are nested within the MOL. Vertical stacking of the integrated *n*/*p* dual WFM is also required during the common gate fabrication process. To form the gate and the contact for the stacked active channels simultaneously, the total height of the structures must be relatively high, and it is therefore necessary to optimize the etching and deposition process with a very high aspect ratio. When compared with sequential CFETs, the monolithic CFET has a compact common gate structure and lower parasitic capacitance and resistance, and it is expected to deliver higher transistor performance [51–55].

In Fig. 5, the advantages and disadvantages of these two CFETs are also summarized briefly. The sequential CFET shows device and material integration flexibility, but suffers from process cost and thermal budget limitations. In contrast, the monolithic CFET shows both cost effectiveness and a self-aligned structure between the top and bottom devices, but it also faces a series of manufacturing process challenges.

### Active layer vertical stacking method

**Figure 6.**
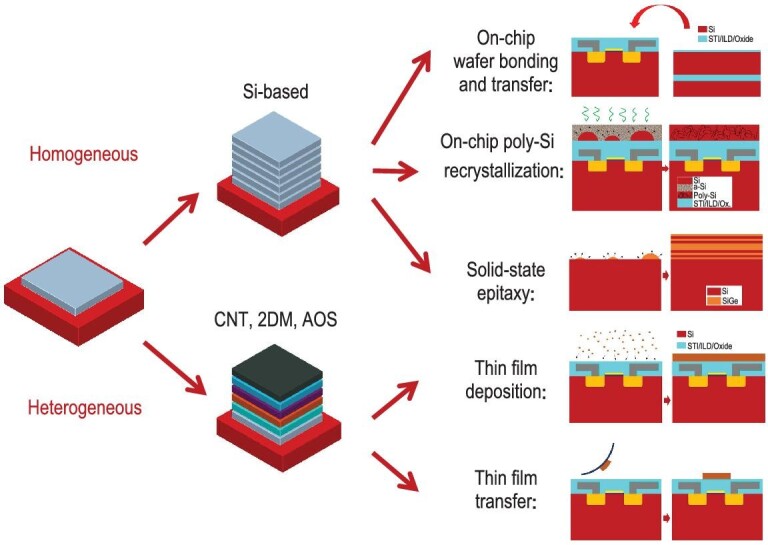
Vertical stacking methods for fabrication of homogeneous and heterogeneous CFETs using Si-based semiconductors and other semiconductors, including CNTs, 2DMs and AOSs.

CFETs can be divided into homogeneous and heterogeneous devices based on their *n*-type and *p*-type channel materials. Development of CFETs using Si-based devices is the main research direction at present. As shown in Fig. 6, the methods for homogeneous integration of the Si channels include on-chip polycrystalline-Si (p-Si) film recrystallization, on-chip wafer bonding and transfer, and solid-state epitaxy.

After formation of the interlayer isolation media, one method involves deposition of α-Si using a far-infrared laser activation process to convert it into p-Si; this is followed by a chemical mechanical polishing (CMP) process to obtain a specific p-Si film thickness on the top layer [56]. Notably, p-Si always contains different grain sizes and multiple defects at the grain boundaries, and this leads to significant transistor performance fluctuations. NARlab used a location-controlled grain process to increase the grain size to 2.56 *$\mu$*m^2^, and the grain distribution and shape were also controllable [57]. Kao *et al.* [58] and other researchers demonstrated additional improved process results. Regardless of the method used to increase the grain size, the grain boundaries and defects are inevitable, and may greatly degrade the resulting device’s performance and reliability, in addition to the integration density of the top transistors.

In contrast, Si active layer transfer based on wafer bonding technology can produce high-quality and uniform single-crystal channels. The major research teams focused on this technology include those at CEA-LETI, IMEC, Intel and STM. After production of the bottom-tier devices, the pre-processed top active layer is transferred onto the wafer with the bottom-tier devices through a low-temperature oxide bonding process. Single-crystal Si layers with specific thicknesses can then be produced using the SmartCut™, etchback or CMP methods. Because the transferred active layer can be pre-processed into single crystals and then subjected to doping and high-temperature annealing steps, the transferred active layer’s quality can reach an optimal state. The only thermal budget requirements for sequential integration of high-quality active layers using the on-chip wafer bonding transfer method relate to the bonding annealing process ($\lt\! 400\, ^{\circ }$C, and the bonding temperature can reach a minimum value of $200\, ^{\circ }$C) [59–61]. Furthermore, the active layer to be transferred in the wafer bonding transfer process can be designed to have various crystal orientations that differ from the underlying materials. The CEA-LETI, IMEC and Intel teams have all successfully bonded and transferred high-quality Si active layers on 300-mm wafers, such as the two-layer Si-based FinFET stack implemented by the IMEC team [59]. Intel has achieved sequential integration of Ge (100) and Si (100), and of Si (100) and GaN on 300-mm wafers [60,61]. For high-performance ICs, vertical stacking of different two-layer Si-based devices provides versatile choices. The devices can be integrated to optimize the *n*- and *p*-type FETs in separate layers, e.g. Si-based devices and their Ge-based counterparts. Si-based devices exhibit useful *n*-type device characteristics with high electron mobility, whereas the Ge-based devices can support higher hole mobility for *p*-type transistors [62]. To enhance the richness of the system performance, different modules can be integrated vertically and suitably advantageous materials can be used for the different modules, e.g. vertically integrated Si-based logic control circuits, high-density SRAM arrays and GaN radio-frequency switching module circuits.

The process of vertical stacking of the active layers by epitaxy can be used for reference for the superlattice epitaxial SiGe_*x*_/Si stacking of the active layers in GAA devices. The difference is that in the GAA devices, SiGe_*x*_/Si acts as a sacrificial layer to isolate the NS channels, and the layer thickness is determined by the thicknesses of the high-κ gate dielectric and metal work function layers. For CFETs, the SiGe_*x*_/Si layer not only plays a role in isolating the NS channels, but also isolates the top and bottom channels, where the layer thickness is determined more strongly by the vertical isolation process used for the SD electrodes, and the layer is usually thicker than that used in GAA devices. Moreover, the active channels grown by epitaxy can present high-quality single-crystal structures without any process interruption. The processing methods for on-chip polycrystalline silicon recrystallization and on-chip wafer bonding transfer are mainly used to produce sequential CFETs, whereas the solid-state epitaxy method is mainly used to fabricate monolithic CFETs. Among these methods, solid-state epitaxy has become the main research and development focus in the industry, with the aim of obtaining high-quality single-crystal channels at low cost.

Given the complex processes required for Si-based homogeneous CFETs and the impact of the thermal budget constraints on the performances of the top-tier devices, heterogeneous CFETs are expected to use emerging materials to maximize the benefits of this new transistor structure. The bottom-layer devices are still the traditional Si-based devices, while the top layers use new device materials with low-temperature processing characteristics, including CNTs, 2DMs and AOSs. These devices demonstrate smaller channel thicknesses and stronger immunity to SCEs, while also maintaining high device performance. The most used on-chip heterojunction integration methods are shown in Fig. 6, and include thin-film deposition and thin-film transfer. A suspension of CNTs can be spin coated onto the substrate and used to prepare on-chip CNT devices. Another method involves direct immersion of the target substrate in the CNT suspension to attach a uniform CNT film (i.e. dip casting), followed by a drying process [65]. Alternatively, pre-deposited and arranged CNTs can be transferred onto a target substrate through a complex transfer process to provide excellent transistor performance [66]. Formation of 2DMs (e.g. MoS_2_ or WSe_2_ layers) on the bottom devices by methods such as CVD, wet transfer [67] and direct mechanical stripping [68] is feasible for heterogeneous integration, but these techniques are not suitable for large-scale ICs. Preparation of 2DMs by MOCVD [69] is becoming the preferred technique in the industry for wafer-scale deposition and provides improved film quality within the processing limits for CFETs.

AOSs are important materials that include IGZO and other amorphous metal oxides. When compared with traditional Si-based semiconductors, AOSs are suitable for processing at lower temperatures, while also maintaining acceptable carrier mobilities [70]. When compared with p-Si devices, AOS-based devices can provide higher carrier mobilities in their amorphous state [71,72]. For bottom-tier devices, IGZO thin films can be formed by either physical sputtering or ALD, and they exhibit high quality and uniformity, making the films suitable for preparation of large-scale ICs based on BEOL 3D integration [73].

### DTCO for CFET

**Figure 7.**
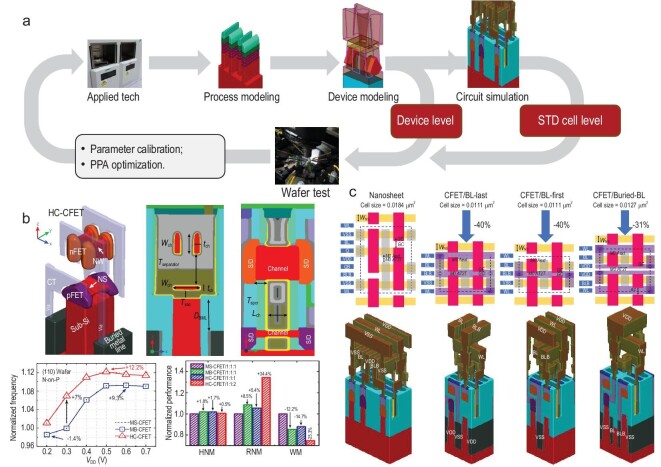
(a) The DTCO process, which includes a device optimization loop and a circuit optimization loop. (b) Novel hybrid-channel CFET (HC-CFET) structure and DTCO evaluation of a ring oscillator circuit and an SRAM cell circuit [63]. (c) Optimization of the CFET layout structure and DTCO evaluation based on parasitic parameter optimization [64].

Beyond device structure and process breakthroughs, CFETs require a full DTCO or STCO to enable the construction of transistors, circuits and even systems at various levels for higher PPA gain. After the 10-nm node, DTCO and advances in electronic design automation are becoming increasingly important for transistors in improving density, enhancing performance and reducing variability [74]. As Fig. 7(a) shows, DTCO includes two optimization approaches: a process-device optimization loop and a process-device-circuit optimization loop. In the process-device optimization loop, universal devices used for large-scale CMOS integration are optimized by targeting device performance parameters such as the device $I\rm _{ON}$, *V*_T_, the *SS*, the off current ($I\rm _{OFF}$) and the *DIBL*. For device performance optimization, new materials or advanced process technologies must be evaluated carefully in the large-scale circuits and not only in individual devices. After device-level testing the device characteristics obtained are compared with the corresponding theoretical results and are used to calibrate the deviations in the emulation environment and then deliver good designs to the actual process manufacturing and circuit design stages.

For IC advancements using the CFET structure, the working DTCO will require further optimization to achieve increased PPA gain with the 3D stackable structures in the vertical direction and the newly introduced materials and technologies. The interface from the CFET to the 3D circuit evaluation is established based on extraction of the device SPICE model and the layout optimization with respect to the SDC and the unit circuits of SRAMs. These are fundamental unit circuits for construction of large-scale ICs and their performances affect the IC performance directly. The typical circuits to be evaluated mainly include the CMOS inverter, the ring oscillator and the SRAM. Based on the calibrated device model obtained, the circuits are fully evaluated in terms of operating speed, energy efficiency and area occupied. With DTCO, innovative CFET structures such as the hybrid-channel CFET (HC CFET; as shown in Fig. 7(b)) [63] can be designed that offer improved SRAM operation margins and speeds for similar power consumption. The layout rule and the impact of parasitic resistance and capacitance on the 3D SRAM based on the CFET is also greatly improved (as shown in Fig. 7(c)) [64].

## NEW PATHS TO A VERTICAL TRANSISTOR

**Figure 8.**
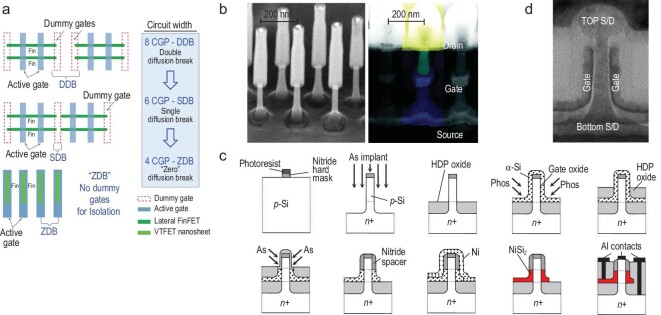
(a) Lateral FinFET/GAAFETs using DDBs or SDBs, which require an increase in the circuit area to accommodate the dummy gates. VGAAFETs can reduce the circuit area by performing STI for a zero-diffusion break (ZDB) solution. (b) TEM image of a Si NW VGAAFET with single-crystal channel growth performed using the vapor-liquid-solid method. (c) Schematic of the Si NW-VGAAFET process flow. (d) TEM images of the Si NW-VGAAFET process flow.

Previous transistor innovations have principally been based on horizontal or lateral conductance channels. Applications using carrier channels and transport paths that are oriented orthogonal to the wafer plane, as in vertical GAAFETs (VGAAFETs), have also been developed, as shown in Fig. 8 [75]. For lateral devices, continuous scaling suggests a continuous reduction in their CGP, which is mainly determined by the *L_G_*, the contact size and the SD spacer width. At present, the trend for *L_G_* reduction is gradually slowing because of the associated SCEs. In the VGAAFET, the *L_G_* is generally determined by the gate material thickness in the vertical direction. Therefore, the device can relax the area penalty and channel size limits involved in achieving higher transistor performance with reduced power consumption [76].

Another major advantage of the use of vertical devices is reduction of the SDC and SRAM cell areas. For FinFETs and lateral GAAFETs, SDBs or DDBs are required to provide isolation [77]. However, VGAAFETs can use STI for circuit isolation to achieve zero-diffusion breaks (ZDBs). Figure 8(a) shows the ZDB scheme for VGAAFETs, which can reduce the device area significantly [78]. When compared with FinFETs, use of vertical devices can achieve a 22.5% reduction in SDC cell area [79]. Moreover, the VGAAFET can be stacked vertically like CFETs using lateral transistors, which can reduce the circuit area further [80].

### Vertical transistors with non-self-aligned gates

Several approaches to VGAAFET fabrication were initially proposed, including both bottom-up (B-U) [81] and top-down (T-D) [82,83] methods to form the vertical channels. The B-U approaches can be divided into two categories according to their process sequences: channel-first and channel-last processes. The vapor-liquid-solid (VLS) method is a B-U channel-first process that uses a metal catalyst and metal-organic vapor-phase epitaxy to grow vertical NWs using various materials, including Si, SiGe, InAs and InP. Figure 8(b) shows a typical Si NW VGAAFET that was fabricated using the VLS process [84]. However, it is difficult to control the NW position and size accurately, and the NW growth is affected significantly by process fluctuations that make it difficult to achieve large-scale mass production.

The B-U channel-last process is similar to the production method used for 3D NAND devices in the memory field [85]. First, multi-layer CVD films are deposited sequentially, followed by photolithography and etching processes to create a cylindrical groove structure with a high aspect ratio. The gate dielectric is then deposited in the groove and materials such as silicon and germanium silicon are grown epitaxially to form the channels. This process enables fabrication of self-aligned gates and well-controlled *L_G_*s, and it is also compatible with the RMG scheme. However, channel formation is dependent on the size of the contact holes. Therefore, when the device size continues to shrink and the aspect ratio of these holes increases, particularly in the case of vertically stacked device structures, it will become increasingly difficult to form high-quality single-crystal channels, which is particularly important for high-performance IC fabrication.

The T-D method, which defines and forms the vertical structures of NWs or NSs using a combination of traditional photolithography and etching processes, is compatible with current mainstream CMOS processes and has thus received significant attention [83,86]. Figure 8(c) shows the process flow for the Si NW VGAAFETs fabricated by the Agency for Science, Technology and Research (A*STAR) of Singapore [75]. IBM has also demonstrated vertical channel NS transistors fabricated using a T-D approach, as shown in Fig. 8(d) [78,87]. These devices show high electrical performance and indicate that VGAAFETs may enable scaling beyond the limits of lateral GAAFETs. However, the device *L_G_* is determined by the thin-film deposition and etching processes, and the process fluctuations and the problems with alignment between the gate and the SD electrodes have not been addressed well to date.

### Vertical devices with self-aligned gates

**Figure 9.**
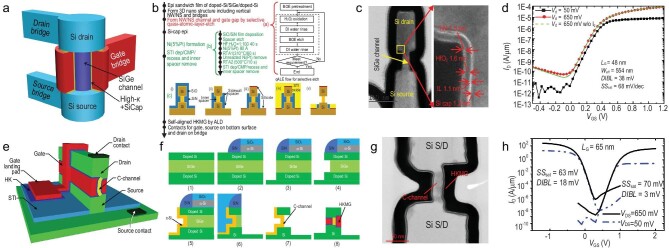
(a) Three-dimensional schematic of a VSAFET. (b) Process flow for VSAFET fabrication, including quasi-atomic layer etch (qALE) and NiPt silicide processes. (c) TEM cross section of a *p*-type NS VSAFET. (d) The transfer curves for an *n*-type NS VSAFET. (e) Three-dimensional schematic of a VCNFET. (f) Schematics of the dual-side process flow for VCNFET fabrication. (g) TEM cross section and (h) transfer curves for a Si VCNFET with a channel thickness of 15 nm and an *L_G_* of 65 nm.

To address the challenges noted above and the process variation issues [88], two new types of VGAAFET, comprising the vertical sandwich GAAFET (VSAFET) [89–91] and the vertical C-shaped-channel NS FET (VCNFET) [92,93], were developed at IMECAS, as shown in Fig. 9. VSAFETs and VCNFETs both exhibit self-aligned HKMG structures and smaller effective-gate-length variations than similar devices obtained by lithography and etching processes because their *L_G_* are determined by epitaxy rather than other factors. VCNFETs can be fabricated while controlling the thicknesses of their NSs easily because they are also formed by the epitaxial growth process. New operational theories based on VSAFETs and VCNFETs, including those for tunneling and ferroelectric VSAFETs [94], may provide greater process feasibility and transistor performance. VGAAFETs also offer new opportunities for 3D integration in dynamic RAM (DRAM) [93] and NOR-type [95] memory applications.

Figure 9(b) shows the process flow for VSAFET fabrication. The process begins with epitaxial growth of Si/SiGe/Si multi-layers on blank wafers. The top and bottom silicon layers can be doped *in situ* by epitaxy or later by ion implantation, enabling them to serve separately as the SD electrodes. The *L_G_* of a VSAFET is mainly determined by the thickness of the SiGe film grown by epitaxy. Next, nano-pillars linked via bridges are patterned by electron-beam lithography and isotropic etching. To form the precisely controlled diameters/thicknesses of the NWs/NSs, the vertical channel, and the lateral gap required for self-aligned gate formation, an isotropic and Si-selective quasi-atomic layer etch (qALE) [96] process is performed. An inner spacer then protects the vertical channel before implementation of the silicide process and the SD deposition process to form *n*-type CMOS devices. TEM cross sections acquired after the ALD-HKMG process are shown in Fig. 9(c). Excellent electrical properties were obtained for both the *p*- and *n*-type CMOS devices, as shown in Fig. 9(d).

Although VSAFETs offer the advantages of self-aligned gates and precise *L_G_* control, the channel dimensions are determined by qALE or by other etching processes, which still suffer from significant fluctuations caused by process deviations. Based on VSAFETs, VCNFETs that offer precise control of both the channel thickness and the *L_G_* have been proposed and fabricated. A 3D schematic and the dual-side process flow diagram for VCNFETs are shown in panels (e) and (f) of Fig. 9, respectively. Using the spacer transfer lithography process, pillars composed of Si/SiGe/Si sandwich films are formed, and the SiGe layer is then partially and selectively etched from the sidewall to form a gate gap, leaving the remaining SiGe as a seed layer. A crystalline Si film called the C-channel is grown epitaxially in the gate gap; this is the key step that enables precise channel thickness control. Another sidewall for the VCNFET can be defined by RIE after the top mandrel is removed. The exposed SiGe seed layer is then etched selectively to release the C-channel and the gate gap is filled with the HKMG stack to form a self-aligned gate with respect to the channel, as illustrated in Fig. 9(g). Beneficial electrical properties were obtained from the VCNFETs, as shown by the transfer curves in Fig. 9(h). The integrated process flow for the VCNFET is compatible with mainstream industrial processes and can easily be extended to 3D stacked devices.

## CONCLUSIONS AND PERSPECTIVES

The introduction of 3D FinFETs and the GAAFET has revolutionized the general transistor platform by enabling continuous development of large-scale ICs through channel structure innovation to provide continued process node scaling with better gate control and improved transistor performance. More advanced transistor structures such as the CFET, 3DS FET and vertical transistor are set to sustain the development of Moore’s law for trailblazing increases in transistor integration density and cell-circuit functionality with innovative 3D integration methods on the transistor level. To enable practical implementation of these new structures in real ICs, the main challenges include the requirements for precise patterning, film deposition and interconnection process technologies, even at the atomic level, which will require major changes to current fabrication methodologies, techniques and equipment. Another critical challenge is the incredible heat generated by the 3D and 3D stacked transistors with high integration numbers in ICs when operating at high speeds, which will require a revolutionary heat dissipation method [97] and structural design from transistor level to chip level. The third critical challenge is the performance and power efficiency of the 3D circuit with the new transistor structure because of the complex 3D routing possibilities and augmented parasitic capacitance, which will require more sophisticated circuit design methodologies and chip architecture innovation.

In the future, to realize higher transistor integration densities and higher computational efficiency, it will be necessary to surpass the thermionic limit of *k_B_T*/*q* (where *k_B_* is the Boltzmann constant and *q* is the electron charge) and the minimum operating voltage of general MOSFETs, which is currently no less than 0.5 V. Submerging the MOSFET into liquid nitrogen, i.e. the cryo-CMOS method [98], may provide a way to reduce operating voltages below 0.2 V. The tunneling MOSFET [99], which operates via quantum tunneling, rather than by general thermal diffusion or drift, can also surpass the thermionic limit in theory and exhibits reduced operating voltages. However, tunneling devices currently suffer from low currents because of their poor quantum tunneling efficiency. A transistor using a Dirac source to generate an energy band tail also provides similar results. Introduction of a ferroelectric film into the gate insulator allows a transistor to exhibit a negative capacitance effect that inherently accumulates surface charges near the channel [100], which also helps this transistor to surpass the thermionic limit and reduce its operating voltage. Ultimately, more advanced ICs can be developed to integrate hundreds of billions of transistors and achieve beyond-CMOS breakthroughs in PPA for further innovations in everyday life.

## References

[1] Peercy PS . The drive to miniaturization. Nature 2000; 406: 1023–6. 10.1038/35023223 10984060

[2] Leiserson CE , Thompson NC, Emer JS et al. There’s plenty of room at the top: what will drive computer performance after Moore’s law? Science 2020; 368: eaam9744. 10.1126/science.aam9744 32499413

[3] Moore GE . Cramming more components onto integrated circuits. Electron Mag 1965; 38: 114–7.

[4] Kelleher AB . Celebrating 75 years of the transistor a look at the evolution of Moore’s law innovation. In: 2022 International Electron Devices Meeting (IEDM), San Francisco, CA, Piscataway, NJ: IEEE Press, 2022, 1.1.1–5. 10.1109/IEDM45625.2022.10019538

[5] Kim K . The smallest engine transforming humanity: the past, present, and future. In: 2021 IEEE International Electron Devices Meeting (IEDM), San Francisco, CA, Piscataway, NJ: IEEE Press, 2021, 1.1.1–8. 10.1109/IEDM19574.2021.9720583

[6] Dennard D . Design of ion-implanted MOSFET’s with very small physical dimensions? IEEE J. Solid-State Circuits 1975; 9: 256–68. 10.1109/JSSC.1974.1050511

[7] Badaroglu M , Xu J, Zhu J et al. PPAC scaling enablement for 5nm mobile SoC technology. In: 2017 47th European Solid-State Device Research Conference (ESSDERC), Leuven, Belgium, Piscataway, NJ: IEEE Press, 2017, 240–3.

[8] Bohr MT and Young IA . CMOS scaling trends and beyond. IEEE Micro 2017; 37: 20–9. 10.1109/MM.2017.4241347

[9] Ferain I , Colinge CA, Colinge JP. Multigate transistors as the future of classical metal-oxide-semiconductor field-effect transistors. Nature 2011; 479: 310–6. 10.1038/nature10676 22094690

[10] IRDS™ 2022: more Moore. International Roadmap for Devices and Systems (IRDS™). 2022 white paper: more than Moore, IEEE, 2022. https://irds.ieee.org/images/files/pdf/2022/2022IRDS_MM.pdf (7 July 2023, date last accessed).

[11] Sayeef S , Kai N, Suman D. The era of hyper-scaling in electronics. Nat Electron 2018; 1: 442–50. 10.1038/s41928-018-0117-x

[12] Veloso A , Huynh-Bao T, Matagne P et al. Nanowire nanosheet FETs for ultra-scaled, high-density logic and memory applications. Solid State Electron 2020; 168: 107736. 10.1016/j.sse.2019.107736

[13] Jeong J , Lee S, Masuoka S. Novel cell architectures with back-side transistor contacts for scaling and performance. In: 2023 IEEE Symposium on VLSI Technology and Circuits (VLSI Technology and Circuits), Kyoto, Japan, Piscataway, NJ: IEEE Press, 2023, 1–2.

[14] Datta S , Chakraborty W and Radosavljevic M. Toward attojoule switching energy in logic transistors. Science 2022; 378: 733–40. 10.1126/science.ade7656 36395210

[15] Liebmann L , Smith J, Chanemougame D et al. CFET design options, challenges, and opportunities for 3D integration. In: 2021 IEEE International Electron Devices Meeting (IEDM), San Francisco, CA, Piscataway, NJ: IEEE Press, 2021, 3.1.1–4. 10.1109/IEDM19574.2021.9720577

[16] Daewon Ha SE . Energy-efficient CMOS scaling for 1nm and beyond. In: 2022 IEEE International Electron Devices Meeting (IEDM), San Francisco, CA, Piscataway, NJ: IEEE Press, 2022, 1–2.

[17] Zhang W , Gao B, Tang J et al. Neuro-inspired computing chips. Nat Electron 2020; 3: 371–82. 10.1038/s41928-020-0435-7

[18] Gonzalez-Zalba M , de Franceschi S, Charbon E et al. Scaling silicon-based quantum computing using CMOS technology. Nat Electron 2021; 4: 872–84. 10.1038/s41928-021-00681-y

[19] Colinge JP , Knoblinger G, Fulde M et al. FinFETs and Other Multi-Gate Transistors. New York: Springer, 2008. 10.1007/978-0-387-71752-4

[20] Saha SK . FinFET Devices for VLSI Circuits and Systems. Boca Raton: CRC Press, 2021.

[21] Kobrinsky M , Silva J, Mannebach E. Novel cell architectures with back-side transistor contacts for scaling and performance. In: 2023 IEEE Symposium on VLSI Technology and Circuits (VLSI Technology and Circuits), Kyoto, Japan, Piscataway, NJ: IEEE Press, 2023, 1–2. 10.23919/VLSITechnologyandCir57934.2023.10185319

[22] Bazizi EM , Pal A, Kim J et al. Materials to systems co-optimization platform for rapid technology development targeting future generation CMOS nodes. IEEE Trans Electron Devices 2021; 5358–63. 10.1109/TED.2021.3076757

[23] Wu SY , Lin C, Chiang M et al. A 7 nm CMOS platform technology featuring 4th generation FinFET transistors with a 0.027 um^2^ high density 6-T SRAM cell for mobile SoC applications. In: 2016 IEEE International Electron Devices Meeting (IEDM), San Francisco, CA, Piscataway, NJ: IEEE Press, 2016, 2.6.1–4. 10.1109/IEDM.2016.7838333

[24] Liu J , Mukhopadhyay S, Kundu A et al. A reliability enhanced 5nm CMOS technology featuring 5th generation finfet with fully-developed EUV and high mobility channel for mobile SoC and high performance computing application. In: 2020 IEEE International Electron Devices Meeting (IEDM), San Francisco, CA, Piscataway, NJ: IEEE Press, 2020, 9.2.1–4. 10.1109/IEDM13553.2020.9372009

[25] Paul A , Bryant A, Hook TB et al. Comprehensive study of effective current variability and MOSFET parameter correlations in 14 nm multi-fin SOI FINFETs. In: 2013 IEEE International Electron Devices Meeting, San Francisco, CA, Piscataway, NJ: IEEE Press, 2013, 13.5.1–4. 10.1109/IEDM.2013.6724625

[26] Zhang Z , Gan W, Li J et al. Scallop-shaped *p*-type finFETs with improved short-channel effects immunity and driving current. Mater Sci Semicond Process 2022; 140: 106337. 10.1016/j.mssp.2021.106337

[27] Cheng K , Seo S, Faltermeier J et al. Bottom oxidation through STI (BOTS) — A novel approach to fabricate dielectric isolated FinFETs on bulk substrates. In: 2014 Symposium on VLSI Technology (VLSI-Technology): Digest of Technical Papers, Honolulu, HI, Piscataway, NJ: IEEE Press, 2014, 1–2. 10.1109/VLSIT.2014.6894390

[28] Zhang Q , Yin H, Luo J et al. FOI FinFET with ultra-low parasitic resistance enabled by fully metallic source and drain formation on isolated bulk-fin. In: 2016 IEEE International Electron Devices Meeting (IEDM), San Francisco, CA, Piscataway, NJ: IEEE Press, 2016, 17.3.1–4. 10.1109/IEDM.2016.7838438

[29] Li M , Fan J, Xu X et al. Investigation on electrostatic discharge robustness of gate-all-around silicon nanowire transistors combined with thermal analysis. IEEE Electron Device Lett 2017; 38: 1653–6. 10.1109/LED.2017.2768484

[30] Ma X , Yin H, Hong P. Gate-all-around silicon nanowire transistors with channel-last process on bulk Si substrate. IEICE Electron 2015; 12: 20150094. 10.1587/elex.12.20150094

[31] Mertens H , Ritzenthaler R, Hikavyy A et al. Gate-all-around MOSFETs based on vertically stacked horizontal Si nanowires in a replacement metal gate process on bulk Si substrates. In: 2016 IEEE Symposium on VLSI Technology, Honolulu, HI, Piscataway, NJ: IEEE Press, 2016, 1–2. 10.1109/VLSIT.2016.7573416

[32] Cheng X , Li Y, Zhao F et al. 4-levels vertically stacked sige channel nanowires gate-all-around transistor with novel channel releasing and source and drain silicide process. Nanomaterials 2022; 12: 889. 10.3390/nano12050889 35269377 PMC8912512

[33] Loubet N , Hook T, Montanini P et al. Stacked nanosheet gate-all-around transistor to enable scaling beyond FinFET. In: 2017 Symposium on VLSI Technology, Piscataway, NJ: IEEE Press, 2017, T230–1.

[34] Bae G , Bae DI, Kang M et al. 3nm GAA technology featuring multi-bridge-channel FET for low power and high performance applications. In: 2018 IEEE International Electron Devices Meeting (IEDM), San Francisco, CA, Piscataway, NJ: IEEE Press, 2018, 28.7.1–4. 10.1109/IEDM.2018.8614629

[35] Li J , Li Y, Zhou N et al. Study of silicon nitride inner spacer formation in process of gate-all-around nano-transistors. Nanomaterials 2020; 10: 793. 10.3390/nano10040793 PMC7221596 32326106

[36] Tian J , He Y, Zhang Q et al. Improving driving current with high-efficiency landing pads technique for reduced parasitic resistance in gate-all-around Si nanosheet devices. ECS J Solid State Sci Technol 2022; 11: 035010. 10.1149/2162-8777/ac5d64

[37] Zhang Q , Gu J, Xu R et al. Optimization of structure and electrical characteristics for four-layer vertically-stacked horizontal gate-all-around Si nanosheets devices. Nanomaterials 2021; 11: 646. 10.3390/nano11030646 33808024 PMC7998492

[38] Gu J , Zhang Q, Wu Z et al. Narrow sub-fin technique for suppressing parasitic-channel effect in stacked nanosheet transistors. IEEE J Electron Devices Soc 2022; 10: 35–9. 10.1109/JEDS.2021.3130123

[39] Zhang J , Frougier J, Greene A et al. Full bottom dielectric isolation to enable stacked nanosheet transistor for low power and high performance applications. In: 2019 IEEE International Electron Devices Meeting (IEDM), San Francisco, CA, Piscataway, NJ: IEEE Press, 2019, 11.6.1–4. 10.1109/IEDM19573.2019.8993490

[40] Yao J , Wei Y, Yang S et al. Record 7(N)+7(P) multiple VTs demonstration on GAA Si nanosheet n/pFETs using WFM-less direct interfacial La/Al-dipole technique. In: 2022 International Electron Devices Meeting (IEDM), Piscataway, NJ: IEEE Press, 2022, 34.2.1–4. 10.1109/IEDM45625.2022.10019361

[41] Liu YC , Tu CT, Tsai CE et al. Highly stacked GeSi nanosheets and nanowires by low-temperature epitaxy and wet etching. IEEE Trans Electron Devices 2021; 68: 6599–604. 10.1109/TED.2021.3110838

[42] Mochizuki S , Colombeau B, Zhang J et al. Structural and electrical demonstration of SiGe cladded channel for PMOS stacked nanosheet gate-all-around devices. In: 2020 IEEE Symposium on VLSI Technology, Honolulu, HI, Piscataway, NJ: IEEE Press, 2020, 1–2. 10.1109/VLSITechnology18217.2020.9265097

[43] Mertens H , Ritzenthaler R, Oniki Y et al. Forksheet FETs with bottom dielectric isolation, self-aligned gate cut, and isolation between adjacent source-drain structures. In: 2022 International Electron Devices Meeting (IEDM), San Francisco, CA, Piscataway, NJ: IEEE Press, 2022, 23.1.1–4. 10.1109/IEDM45625.2022.10019497

[44] Tu CT , Hsieh WH, Huang BW et al. Experimental demonstration of treefets combining stacked nanosheets and low doping interbridges by epitaxy and wet etching. IEEE Electron Device Lett 2022; 43: 682–5. 10.1109/LED.2022.3159268

[45] Cao L , Zhang Q, Luo Y et al. Novel channel-first fishbone FETs with symmetrical threshold voltages and balanced driving currents using single work function metal process. IEEE Trans Electron Devices 2022; 69: 5971–7. 10.1109/TED.2022.3206179

[46] Cao L , Zhang Q, Yao J et al. Investigation of fabricated CMOS fishboneFETs and treeFETs with strained SiGe nano-fins on bulk-Si substrate. IEEE Electron Device Lett 2023; 44: 1396–9. 10.1109/LED.2023.3294545

[47] Li X , Zhu H, Gan W et al. A three-dimensional simulation study of the novel comb-like-channel field-effect transistors for the 5-nm technology node and beyond. IEEE Trans Electron Devices 2022; 69: 4786–90. 10.1109/TED.2022.3188589

[48] Ryckaert J , Na MH, Weckx P et al. Enabling sub-5nm CMOS technology scaling thinner and taller fin. In: 2019 IEEE International Electron Devices Meeting (IEDM), San Francisco, CA, Piscataway, NJ: IEEE Press, 2019, 29.4.1–4. 10.1109/IEDM19573.2019.8993631

[49] Yang S , Schuddink P, Garcia B. PPA and scaling potential of backside power options in N2 and A14 nanosheet technolog. In: 2023 IEEE Symposium on VLSI Technology and Circuits (VLSI Technology and Circuits), Kyoto, Japan, Piscataway, NJ: IEEE Press, 2023, 1–2.

[50] Batude P , Fenouillet-Beranger C, Pasini L et al. 3DVLSI with CoolCube process: an alternative path to scaling. In: 2015 Symposium on VLSI Technology (VLSI Technology), Kyoto, Japan, Piscataway, NJ: IEEE Press, 2015, T48–9. 10.1109/VLSIT.2015.7223698

[51] Hong TZ , Chang WH, Agarwal A et al. First demonstration of heterogenous complementary FETs utilizing low-temperature (200 ^○^C) hetero-layers bonding technique (LT-HBT). In: 2020 IEEE International Electron Devices Meeting (IEDM), San Francisco, CA, Piscataway, NJ: IEEE Press, 2020, 15.5.1–4. 10.1109/IEDM13553.2020.9372001

[52] Yang M , Chan V, Chan K et al. Hybrid-orientation technology (hot): opportunities and challenges. IEEE Trans Electron Devices 2006; 53: 965–78. 10.1109/TED.2006.872693

[53] Huang CY , Dewey G, Mannebach E et al. 3-D self-aligned stacked NMOS-on-PMOS nanoribbon transistors for continued moore’s law scaling. In: 2020 IEEE International Electron Devices Meeting (IEDM), San Francisco, CA, Piscataway, NJ: IEEE Press, 2020, 20.6.1–4. 10.1109/IEDM13553.2020.9372066

[54] Chang SW , Sung PJ, Chu TY et al. First demonstration of CMOS inverter and 6T-SRAM based on GAA CFETs structure for 3D-IC applications. In: 2019 IEEE International Electron Devices Meeting (IEDM), San Francisco, CA, Piscataway, NJ: IEEE Press, 2019, 11.7.1–4. 10.1109/IEDM19573.2019.8993525

[55] Radosavljević M , Huang CY, Rachmady W et al. Opportunities in 3-D stacked CMOS transistors. In: 2021 IEEE International Electron Devices Meeting (IEDM), San Francisco, CA, Piscataway, NJ: IEEE Press, 2021, 34.1.1–4. 10.1109/IEDM19574.2021.9720633

[56] Yang CC , Hsieh TY, Huang PT et al. Location-controlled-grain technique for monolithic 3D BEOL FinFET circuits. In: 2018 IEEE International Electron Devices Meeting (IEDM), San Francisco, CA, Piscataway, NJ: IEEE Press, 2018, 11.3.1–4. 10.1109/IEDM.2018.8614708

[57] Hsieh PY , Chang YJ, Chen PJ et al. Monolithic 3D BEOL FinFET switch arrays using location-controlled-grain technique in voltage regulator with better FOM than 2D regulators. In: 2019 IEEE International Electron Devices Meeting (IEDM), San Francisco, CA, Piscataway, NJ: IEEE Press, 2019, 3.1.1–4. 10.1109/IEDM19573.2019.8993441

[58] Kao MH , Chen WH, Hou PC et al. Flexible and transparent BEOL monolithic 3DIC technology for human skin adaptable internet of things chips. In: 2020 IEEE Symposium on VLSI Technology, Honolulu, HI, Piscataway, NJ: IEEE Press, 2020, 1–2. 10.1109/VLSITechnology18217.2020.9265079

[59] Vandooren A , Franco J, Wu Z et al. First demonstration of 3D stacked finfets at a 45nm fin pitch and 110nm gate pitch technology on 300mm wafers. In: 2018 IEEE International Electron Devices Meeting (IEDM), San Francisco, CA, Piscataway, NJ: IEEE Press, 2018, 7.1.1–4. 10.1109/IEDM.2018.8614654

[60] Rachmady W , Agrawal A, Sung S et al. 300mm heterogeneous 3D integration of record performance layer transfer germanium PMOS with silicon NMOS for low power high performance logic applications. In: 2019 IEEE International Electron Devices Meeting (IEDM), San Francisco, CA, Piscataway, NJ: IEEE Press, 2019, 29.7.1–4. 10.1109/IEDM19573.2019.8993626

[61] Then HW , Dasgupta S, Radosavljevic M et al. 3D heterogeneous integration of high performance high-K metal gate GaN NMOS and Si PMOS transistors on 300mm high-resistivity Si substrate for energy-efficient and compact power delivery, RF (5G and beyond) and SoC applications. In: 2019 IEEE International Electron Devices Meeting (IEDM), San Francisco, CA, Piscataway, NJ: IEEE Press, 2019, 17.3.1–4. 10.1109/IEDM19573.2019.8993583

[62] Zhang R , Tang X, Yu X et al. Aggressive EOT scaling of Ge pMOSFETs with HfO_2_/AlO_*x*_/GeO_*x*_ gate-stacks fabricated by ozone postoxidation. IEEE Electron Device Lett 2016; 37: 831–4. 10.1109/LED.2016.2572731

[63] Luo Y , Zhang Q, Cao L et al. Investigation of novel hybrid channel complementary fet scaling beyond 3-nm node from device to circuit. IEEE Trans Electron Devices 2022; 69: 3581–8. 10.1109/TED.2022.3176843

[64] Luo Y , Cao L, Zhang Q et al. Layout optimization of complementary FET 6T-SRAM cell based on a universal methodology using sensitivity with respect to parasitic values. IEEE Trans Electron Devices 2022; 69: 6095–101. 10.1109/TED.2022.3207972

[65] Zhong D , Xiao M, Zhang Z et al. Solution-processed carbon nanotubes based transistors with current density of 1.7 ma/m and peak transconductance of 0.8 ms/m. In: 2017 IEEE International Electron Devices Meeting (IEDM), San Francisco, CA, Piscataway, NJ: IEEE Press, 2017, 5.6.1–4. 10.1109/IEDM.2017.8268335

[66] Liu L , Han J, Xu L et al. Aligned, high-density semiconducting carbon nanotube arrays for high-performance electronics. Science 2020; 368: 850–6. 10.1126/science.aba5980 32439787

[67] Tong L , Wan J, Xiao K et al. Monolayer transistors at wafer scales. Nat Electron 2023; 6: 37–44.

[68] Pan Y , Yin H, Huang K et al. Novel 10-nm gate length MoS_2_ transistor fabricated on Si fin substrate. IEEE J Electron Devices Soc 2019; 7: 483–8. 10.1109/JEDS.2019.2910271

[69] Xiang D , Liu T. Monolayer transistors at wafer scales. Nat Electron 2021; 4: 868–9. 10.1038/s41928-021-00694-7

[70] Kamiya T , Nomura K, Hosono H. Present status of amorphous In–Ga–Zn–O thin-film transistors. Sci Technol Adv Mate 2010; 11: 044305. 10.1088/1468-6996/11/4/044305 PMC5090337 27877346

[71] Chang SW , Lu TH, Yang CY et al. First demonstration of heterogeneous IGZO/Si CFET monolithic 3D integration with dual workfunction gate for ultra low-power SRAM and RF applications. In: 2021 IEEE International Electron Devices Meeting (IEDM), San Francisco, CA, Piscataway, NJ: IEEE Press, 2021, 34.4.1–4. 10.1109/IEDM19574.2021.9720675

[72] An R , Li Y, Tang J et al. A hybrid computing-in-memory architecture by monolithic 3D integration of BEOL CNT/IGZO-based CFET logic and analog rram. In: 2022 International Electron Devices Meeting (IEDM), San Francisco, CA, Piscataway, NJ: IEEE Press, 2022, 18.1.1–4. 10.1109/IEDM45625.2022.10019473

[73] Allemang CR , Cho TH, Trejo O et al. High–performance zinc tin oxide TFTs with active layers deposited by atomic layer deposition. Adv Electron Mater 2020; 6: 2000195. 10.1002/aelm.202000195

[74] Moroz V , Lin XW, Asenov P et al. DTCO launches Moore’s law over the feature scaling wall. In: 2020 IEEE International Electron Devices Meeting (IEDM), San Francisco, CA, Piscataway, NJ: IEEE Press, 2020, 41.1.1–4. 10.1109/IEDM13553.2020.9372010

[75] Chen Z , Singh N, Kwong D. Vertical silicon nanowire MOSFET with a fully-silicided (FUSI) NiSi_2_ gate. World Acad Eng Technol 2011; 81: 681–3.

[76] Veloso V , Sanchez E, Brus S et al. Vertical nanowire FET integration and device aspects. ECS trans 2016; 72: 31–42. 10.1149/07204.0031ecst

[77] Pal A , Bazizi EM, Jiang L et al. Self-aligned single diffusion break technology optimization through material engineering for advanced CMOS nodes. In: 2020 International Conference on Simulation of Semiconductor Processes and Devices (SISPAD), Kobe, Japan, Piscataway, NJ: IEEE Press, 2020, 307–10. 10.23919/SISPAD49475.2020.9241625

[78] Jagannathan H , Anderson B, Sohn CW et al. Vertical-transport nanosheet technology for CMOS scaling beyond lateral-transport devices. In: 2021 IEEE International Electron Devices Meeting (IEDM), San Francisco, CA, Piscataway, NJ: IEEE Press, 2021, 26.1.1–4. 10.1109/IEDM19574.2021.9720561

[79] Song T . Opportunities and challenges in designing and utilizing vertical nanowire FET (V-NWFET) standard cells for beyond 5 nm. IEEE Trans Nanotechnology 2019; 18: 240–51. 10.1109/TNANO.2019.2896362

[80] Song T . Many-tier vertical GAAFET (V-FET) for ultra-miniaturized standard cell designs beyond 5 nm. IEEE Access 2020; 8: 149984–98. 10.1109/ACCESS.2020.3015596

[81] Oh SH , Hergenrother J, Nigam T et al. 50 nm vertical replacement-gate (VRG) pMOSFETs. In: 2000 IEEE International Electron Devices Meeting (IEDM), San Francisco, CA, Piscataway, NJ: IEEE Press, 2000, 65–8.

[82] Maheshwaram S , Manhas SK, Kaushal G et al. Vertical silicon nanowire gate-all-around field effect transistor based nanoscale cmos. IEEE Electron Device Lett 2011; 32: 1011–3. 10.1109/LED.2011.2157076

[83] Gandhi R , Chen Z, Singh N et al. Cmos-compatible vertical-silicon-nanowire gate-all-around p-type tunneling FETs with ≤50-mv/decade subthreshold swing. IEEE Electron Device Lett 2011; 32: 1504–6. 10.1109/LED.2011.2165331

[84] Berg M , Persson KM, Kilpi OP et al. Self-aligned, gate-last process for vertical InAs nanowire MOSFETs on Si. In: 2015 IEEE International Electron Devices Meeting (IEDM), Washington, DC, Piscataway, NJ: IEEE Press, 2015, 31.2.1–4. 10.1109/IEDM.2015.7409806

[85] Kar GS , Van den bosch G, Cacciato A et al. Novel Bi-layer poly-silicon channel vertical flash cell for ultrahigh density 3D SONOS NAND technology. In: 2011 3rd IEEE International Memory Workshop (IMW), Monterey, CA, Piscataway, NJ: IEEE Press, 2011, 1–4. 10.1109/IMW.2011.5873209

[86] Larrieu G and Han XL . Vertical nanowire array-based field effect transistors for ultimate scaling. Nanoscale 2013; 5: 2437–41. 10.1039/c3nr33738c 23403487

[87] Tsutsui G , Song S, Strane J et al. Hardware based performance assessment of vertical-transport nanosheet technology. In: 2022 International Electron Devices Meeting (IEDM), San Francisco, CA, Piscataway, NJ: IEEE Press, 2022, 34.4.1–4. 10.1109/IEDM45625.2022.10019393

[88] Kuhn KJ , Giles MD, Becher D et al. Process technology variation. IEEE Trans Electron Devices 2011; 58: 2197–208. 10.1109/TED.2011.2121913

[89] Yin X , Zhang Y, Zhu H et al. Vertical sandwich gate-all-around field-effect transistors with self-aligned high-k metal gates and small effective-gate-length variation. IEEE Electron Device Lett 2020; 41: 8–11. 10.1109/LED.2019.2954537

[90] Li C , Zhu H, Zhang Y et al. First demonstration of novel vertical gate-all-around field-effect-transistors featured by self-aligned and replaced high-metal gates. Nano Lett 2021; 21: 4730–7. 10.1021/acs.nanolett.1c01033 34038143

[91] Zhang Y , Ai X, Yin X et al. Vertical sandwich GAA FETs with self-aligned high-k metal gate made by quasi atomic layer etching process. IEEE Trans Electron Devices 2021; 68: 2604–10. 10.1109/TED.2021.3072879

[92] Xiao ZR , Wang Q, Zhu HL et al. Vertical C-shaped-channel nanosheet FETs featured with precise control of both channel-thickness and gate-length. IEEE Electron Device Lett 2022; 43: 1183–6. 10.1109/LED.2022.3187006

[93] Xiao ZR , Zhu HL, Wang Q et al. Vertical n-type and p-type nanosheet FETs with C-shaped channel. IEEE Trans Electron Devices 2023; 70: 1380–5. 10.1109/TED.2023.3239048

[94] Huang W , Zhu H, Zhang Y et al. Ferroelectric vertical gate-all-around field-effect-transistors with high speed, high density, and large memory window. IEEE Electron Device Lett 2022; 43: 25–8. 10.1109/LED.2021.3126771

[95] Huang W , Zhu H, Li J et al. A novel 3D nor flash with single-crystal silicon channel: devices, integration, and architecture. IEEE Electron Device Lett 2022; 43: 1874–7. 10.1109/LED.2022.3211174

[96] Yin X , Zhu H, Zhao L et al. Study of isotropic and Si-selective quasi atomic layer etching of Si_1−*x*_Ge_*x*_. ECS J Solid State Sci Technol 2020; 9: 034012. 10.1149/2162-8777/ab80ae

[97] Lundstrom M and Alam M . Moore’s law: the journey ahead. Science 2022; 378: 722–3. 10.1126/science.ade2191 36395227

[98] Chakraborty W , Aabrar KA, Gomez J et al. Characterization and modeling of 22 nm FDSOI cryogenic RF CMOS. IEEE J Exploratory Solid-State Comput. Devices Circuits 2021; 7: 184–92. 10.1109/JXCDC.2021.3131144

[99] Qiu Y , Wang R, Huang Q et al. A comparative study on the impacts of interface traps on tunneling FET and MOSFET. IEEE Trans Electron Devices 2014; 61: 1284–91. 10.1109/TED.2014.2312330

[100] Zhang Z , Tian G, Huo J et al. Recent progress of hafnium-oxide based ferroelectric devices for advanced circuit applications. Sci China Inf Sci 2023; 66: 200405. 10.1007/s11432-023-3780-7

